# Mindfulness in Action: How Innovative and Inclusive Climates Affect Proactive Behaviors Through Mental Toughness

**DOI:** 10.1002/pchj.70097

**Published:** 2026-04-29

**Authors:** Xuemin Han, Huan Zhou, Guoqiang Zeng, Yicen Han

**Affiliations:** ^1^ Sports Department Hainan University, School of Tropical Agriculture and Forestry (SARA, SRR) Danzhou Hainan Province China; ^2^ School of Business Administration, Southwestern University of Finance and Economics Chengdu Sichuan Province China; ^3^ China Center for Behavioral Economics and Finance, Southwestern University of Finance and Economics Chengdu Sichuan Province China; ^4^ Department of Respiratory and Critical Care Medicine Chengdu Second People's Hospital Chengdu Sichuan Province China

**Keywords:** inclusive climates, innovative climates, mental toughness, mindfulness, proactive behaviors

## Abstract

In competitive sports, formal athletes' proactive behaviors are crucial for their on‐stream performance. Recent studies suggest mindfulness training and mental toughness can enhance athletes' performance, but their impact on sustained proactive behaviors remains unclear. Considering the team's psychological climate, our study explores how innovative and inclusive climates moderate the relationship between mindfulness, mental toughness, and proactive behaviors. A cross‐sectional survey of 483 formal athletes in China was conducted, employing structural equation modeling to test hypotheses. Results indicate that mindfulness enhances proactive behaviors indirectly through mental toughness. An innovative climate positively moderates the indirect relationship and reverses in direct, while an inclusive climate does not. The study contributes to sports psychology by establishing an integrated model of mindfulness‐driven proactive behavior, revealing differentiated moderation paths through mental toughness, and providing practical insights for optimizing team climates to foster proactive behaviors.

## 1 Introduction

In competitive sports, formal athletes' proactive behaviors (e.g., proactively adjusting training plans and anticipating opponents' tactical changes) have gradually become an essential psychological factor in determining their performance in the game (Hsiao and Wang 2020; Lu 2020; Oliver et al. 2010). Such behaviors are observed across different types of sport, including open versus closed skills, individual versus team sports, and contact versus noncontact sports (Benson et al. 2014; Li et al. 2024).

For instance, in open skill sports (e.g., soccer, basketball), athletes' proactive behaviors are developed through ordinary unpredictable external stimuli (e.g., opponents' movements, environmental conditions). External stimuli raise the demand on athletes to pay extra attention to situational awareness. In contrast, closed skill sports (e.g., swimming, gymnastics) involve more controlled, predictable environments, where athletes' proactive behavior primarily stems from technical awareness of changes in a stable setting (Macali et al. 2025). The different stimuli for proactive behaviors in individual and team sports have a varied origin of awareness. In individual sports (e.g., tennis, athletics), it stems from intrinsic motivation, which is the awareness of personal goals behind. In contrast, team sports (e.g., football, rugby) require athletes to coordinate their targets with others; awareness of team dynamics is crucial (Oliver et al. 2010). Lastly, in contact sports (e.g., rugby, boxing), proactive behaviors are developed through the awareness of countering physical damage. Conversely, in noncontact sports (e.g., tennis, cycling), awareness of maintaining sustained concentration and managing psychological pressure is a major incentive.

As mentioned above, athletes' awareness, as a behavioral incentive for proactive behaviors, is an important prerequisite. To enhance such awareness, mindfulness training, raising awareness through intentionally paying attention, has been gradually introduced to promote athletes' awareness regulation and emotional control in their behaviors (Pineau et al. 2014). Shaabani et al. (2020) and Terzioğlu and Çakir‐Çelebi (2025) supported the idea that mindfulness could increase athletes' awareness of target behaviors and reduce their pressure during behavioral engagement. Mahalingam (2024), from the perspective of pressure, indicated that mindfulness training reduces behavioral tension and anxiety but lacks significant evidence for activating their behavior. Thus, the translation of mindfulness incentive to proactive behaviors remains unexplored.

Madrigal et al. (2013) introduced a clue linking individuals' mindfulness to their behavior, which they explained as mental toughness. Mental toughness refers to a set of attributes that enable an individual to stay focused, motivated, and persistent in the face of pressure (Gucciardi et al. 2021). Compared to mental, which involves resilience recovery from adversity, trauma, or stress, mental toughness reduces the complexity of decision‐making and behavioral engagement in individuals (Nien et al. 2020). Mental toughness enhances mindfulness on the one hand and focuses their awareness on their behaviors on the other (Chen et al. 2024; Galily et al. 2024). To determine the success of mental toughness in sport, we found that it incentivized real‐time awareness (Mahalingam 2024), promoted emotional regulation (Martinent et al. 2015), and revived behavioral correlates (Gucciardi et al. 2015) for athletes. Thus, a clear incentive mechanism of mental toughness translation needs further investigation.

However, to explicitly explain the above incentive mechanism and its dynamics, one needs to consider the external factors surrounding athletes. Formal athletes surrounded by their teammates and workmates are organized within a specific psychological climate. According to the theory of organizational psychological climate, organizational members act their behaviors based on their characterized understanding of surroundings (Baltes et al. 2009). As the organizational members of athletic teams, athletes prompting their diversified understanding of different psychological climates tend to incentivize or inhibit target behavior (Chu et al. 2018; Wilson 2018).

A transferable linkage of such organizational climate into sports psychology is the research attempt at motivational climate (Lacerda et al. 2021; Olympiou et al. 2008; Stanger et al. 2018). Motivational climates, progressing in educational psychology and sports psychology, explained the contextual incentive on athletes' behaviors among competitors and teammates (Robinson 2023). However, ignoring the views of seeing formal athletes as organizational members thereby limits their behavioral incentives to a competitor's scene. Surprisingly, some new attempts, transferring different states of organizational climate into a formal athletic team, provided us with a specific look (Meng et al. 2024; Scott et al. 2025).

Under the construct of motivational climates, the specific meaning of psychological climate raises athletes' behavioral withdrawal or engagement (Robinson 2023). The specific organizational psychological climates (innovative vs. inclusive) provided us with a deeper understanding of these contextual incentives (Kim et al. 2024; Zahoor et al. 2024). An innovative climate refers to a team culture that supports the new tactics and helps athletes overcome pressure, thereby motivating exploratory behavior (e.g., experimenting with unconventional techniques) by providing “freedom of action” (Kim et al. 2024). An inclusive climate emphasizes fairness, belonging, and psychological safety among members (Nishii 2013; Zahoor et al. 2024), thereby creating psychological safety for releasing pressure. So, as contextual factors, both types of climates have been shown to validate athletes' direct interpretations of their target behavior, and at the same time influence the pressure they are under. Thus, considering the climate's effect, a moderated mediation incentive mechanism remains unexplored.

Our research aims to fill the research gaps mentioned: (1) the incentive mechanism between mindfulness and proactive behaviors, (2) the mediating role of mental toughness, and (3) the interactions and differences of innovative and inclusive climates on the incentive mechanism.

**FIGURE 1:**
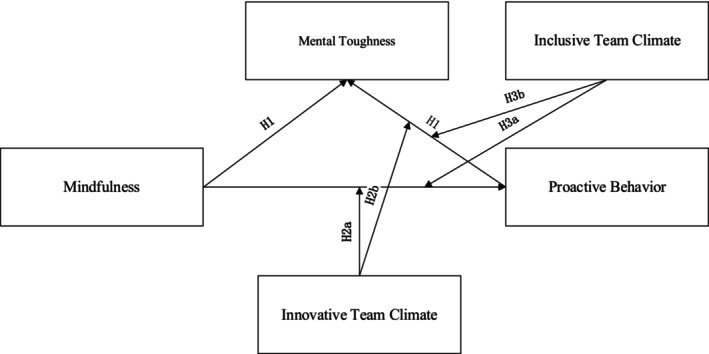
Hypothesis model.

This research fills identified research gaps by proposing a unique model, shown in Figure 1. By highlighting mental toughness as a translating role, we explain the incentive mechanism of proactive behavior. Further elucidating this process through resource conservation theory (Hobfoll 1989), we explain how mental toughness helps conserve and enhance the psychological resources required for proactive behavior. Additionally, we introduce situational strength theory (Meyer et al. 2020) to explore how innovative and inclusive team climates moderate the above relationship. By integrating these theories, we construct a new framework that not only elucidates the psychological processes but also explains how external psychological climate shapes formal athletes' behavior. This combination of an internal mechanism (mental toughness) and an external moderating variable (psychological climates) gives our model a special advantage in addressing existing research gaps. As for the procedures, we conducted a cross‐sectional survey of 483 formal athletes in the Chinese mainland, which was under the required sample size. As for analytic methods, we used structural equation modeling (SEM) with process macros to test hypotheses.

The contribution of this study is threefold. On the theoretical level, it is the first time to establish an integrated model of mindfulness‐driven proactive behavior in sports psychology (Sawyer et al. 2022; Shahbaz and Parker 2022). We break through the limitations of traditional research on the “passive adaptive function” of mindfulness and reveal the differentiated regulation paths of mental toughness and innovative and inclusive climate (Madrigal et al. 2013; Olympiou et al. 2008). It has deepened our theoretical understanding of the psychological‐level team climate (Galily et al. 2024; Nien et al. 2020). As for practice, this study provided a basis for coaching teams to design an integration program containing mindfulness building, mental strengthening, and climate adaptation (Madrigal et al. 2013). For example, formalizing athletes' proactive behavior and relaxing their competition anxiety through mental toughness could lead to a tactical breakthrough in field performance (Wilson et al. 2006). At the last mechanism level, through quantifying the moderating effect, we clarify the dynamic matching logic of psychological climate to contextual empowerment and lay the methodological foundation for subsequent longitudinal intervention studies. The hypothesis model is shown in Figure 1 below.

## 2 Theoretical Underpinning and Hypothesis Development

Athletes' mindfulness could be explained as a nonjudgmental attention to the experience of the present movement (Brown and Ryan 2003). As a positive psychological resource for athletes' behavior, it reduces cognitive fixation (e.g., over‐reflecting on mistakes) and emotional depletion (e.g., pre‐game anxiety) (Gardner and Moore 2019; Nien et al. 2020; Pineau et al. 2014). Through attentional regulation and emotional acceptance, mindfulness is perceived as providing cognitive space for proactive behaviors (Petrou et al. 2024).

Based on Hobfoll (1989) conservation of resources theory (COR), positive thinking drives individuals' behavior through a dual pathway. First, resource accumulation, which includes the conservation of mental energy by filtering irrelevant stimuli (e.g., spectator distractions). Second, resource transformation, which includes the conversion of the conserved energy into goal‐directed actions (e.g., proactive analysis of video footage after a match). For example, athletes with high levels of mindfulness are more likely to attribute failures to improvement opportunities than to self‐denial, a cognitive reframing that significantly enhances the frequency of their tactical adjustments (Piasecki et al. 2025). However, the driving effect of mindfulness on proactive behavior does not exist in isolation but is highly dependent on the organizational climate within the team.

According to situational strength theory, organizational climate acted as a strong situation, shaping individual behaviors through norm consistency and outcome predictability (Meyer et al. 2020). Specifically, an innovative team climate provided a context of support on risk‐taking and tolerance for failure which reinforced thoughts driving athletes' positive mental resources and competitive behaviors by releasing “freedom of action” signals, while an inclusive team climate emphasizing mental safety and collaborative behaviors enhances thoughts of team adaptive behaviors by decreasing social threats (e.g., blaming for mistakes). While the independent effects of the two types of team climate have been partially validated, their synergistic or conflicting effects remain to be explored.

To explain this, our study introduced the regulatory focus theory (Higgins 1997) and mental toughness (Gucciardi et al. 2015), which proposed that athlete behavior is driven by two types of motivation: competitive orientations (pursuing to be better among teammates, e.g., individual breakthroughs) versus collaborative orientations (seeking mutually profitable with teammates, e.g., teamwork strategy) (Hogan 2020; Thornton et al. 2011). An innovative climate motivates positive resources to invest in proactive behaviors by activating competitive orientations, while an inclusive climate safeguards psychological safety and decreased social threats to maintain behavioral continuity by reinforcing collaborative orientations. This theoretical framework not only reveals the dynamic matching logic between the climate and psychological resources but also provides support for predicting behavioral patterns in complex situations.

### 2.1 Mindfulness, Mental Toughness, and Proactive Behaviors

Mindfulness, as a metacognitive self‐regulatory ability, is centered on helping athletes maintain an efficient allocation of mental resources in high‐pressure environments through nonjudgmental attention regulation. According to Resource Conservation Theory (Hobfoll 1989), individuals face a continuous depletion of psychological resources during athletics, which includes multiple challenges such as coping with competition stress (Sharpe et al. 2024), dealing with immediate decision‐making (Brown and Stenling 2025), and managing emotional swings (Fritsch et al. 2024).

Mindfulness training significantly reduces mental resource depletion due to rumination thinking (e.g., repeatedly dwelling on missed movements) and anticipatory anxiety (e.g., excessive worry about the outcome of the competition) by fostering athletes' openness to awareness of present‐moment experiences, a mechanism supported by a large body of empirical research (Fritsch et al. 2024; Shaabani et al. 2020; Terzioğlu and Çakir‐Çelebi 2025; Ticklay and Jones 2024; Zhang et al. 2025). This reflected the COR theory of how athletes actively allocate and conserve their limited mental resources to mobilize their behavior.

More specifically, when athletes use a nonjudgemental attention facing their competition, their preserved mental resources can be more effectively invested in goal‐directed behaviors, such as proactively analyzing technical videos, adjusting training plans, or innovating tactical strategies (Zhang et al. 2025). Existing studies have shown that after mindfulness interventions, athletes not only show better stress adaptation but also a significant increase in the frequency of their behaviors of actively seeking technical improvements and tactical innovations (Shaabani et al. 2020; Zhang et al. 2025).

Further, the role of mindfulness is achieved through the accumulation of cognitive resources, called mental toughness. Mental toughness, as an individual's key mental resource, maintains the balance of stress growth of athletes (Gucciardi et al. 2015). It plays an important role in maintaining the stress raised by growing mindfulness to the target behavior (Martinent et al. 2015), which aligns with the conservation theory of protecting limited mental resources from overload stress (Hobfoll 2011). As for mindfulness training, it could enable athletes to focus on the right now situation and be tough in a resilient mental state (Gucciardi et al. 2021). The feedback in a more constructive cognition could enhance their mental resources growth to target behavior (Martinent et al. 2015; Wagstaff et al. 2012). Thus, our study proposed H1:

*Mindfulness positively enhances athletes' proactive behaviors mediating from mental toughness*.

### 2.2 Innovative Team Climate for Proactive Behaviors

According to individuals' regulatory focus, innovative team climates provide critical environmental empowerment for their proactive behaviors by drawing their focus and tolerance of risk‐taking and failure (Kang et al. 2016). As situational strength theory said (Meyer et al. 2020), when teams explicitly communicate the behavioral norm that “experimentation with new strategies is encouraged,” this creates a strong contextual signal that significantly lowers athletes' psychological threshold for innovative risk‐taking (Brown and Bray 2017). Proactive behavior reinforced by mental toughness—such as resistance to facing pressure and openness to potential failure—is further activated in this highly innovative climate (Gucciardi et al. 2021; Kim et al. 2024; Oliver et al. 2010). The underlying mechanism lies in the fact that the innovative climate, by reinforcing the facilitative focus in competitive orientations (Higgins 1997; Thornton et al. 2011), directs the psychological resources accumulated by proactivity towards individual breakthroughs (e.g., developing more threatening offense than other teammates) rather than only for minimizing potential losses (e.g., error avoidance).

This situational‐dependent effect reveals two parts of the shaping power on athletes' proactive behavior. One is the innovative climate adjusts the influence from mindfulness to behavioral potential, and the other is the extra way of mental toughness mediation. Therefore, we presented H2 that:

*Innovative team climate positively moderates the relationship between mindfulness and proactive behavior* (*a*) *and the relationship between mental toughness and proactive behavior* (*b*).

### 2.3 Inclusive Team Climate for Proactive Behaviors

As the same theoretical supports, inclusive climate primarily fosters psychological safety, fairness, and a sense of belonging among team members (Li et al. 2022; Nishii 2013; Zahoor et al. 2024). It transforms members' focus on perceived social threats, such as fear of blame for mistakes or rejection of divergent ideas, thereby lowering defensive reactions and potentially freeing psychological resources for behavior (Nishii and Rich 2014). The reduction, raised by the psychological situation, or the team environment, of social threat cues within an inclusive climate likely mitigates the perceived interpersonal risks associated with proactive behaviors, making athletes more willing to translate mindful awareness into observable proactive efforts (Van Yperen et al. 2021).

Mental toughness equips athletes to persist under pressure and manage setbacks (Gucciardi et al. 2015). An inclusive climate, by reinforcing collaborative orientations and reducing the fear of negative social consequences for taking initiative (Hogan 2020; Nishii and Rich 2014), provides the psychological safety net that encourages mentally tough athletes to leverage their awareness towards proactive behaviors, such as experimenting with new techniques in practice or offering constructive feedback, rather than solely focusing on personal endurance or error avoidance.

While an inclusive climate safeguards against social threats and fosters belonging, its primary regulatory focus aligns with collaborative orientations aimed at maintaining harmony and psychological safety (Hogan 2020; Li et al. 2022). In contrast, an innovative climate actively promotes competitive orientations centered on individual breakthroughs and exploration by explicitly endorsing risk‐taking and tolerating failure (Kim et al. 2024). Proactive behaviors often require not just safety but also explicit permission and encouragement to challenge norms and take calculated risks—a signal more powerfully sent by an innovative climate than by an inclusive one focused on security and cohesion (Fu et al. 2025; Kim et al. 2024; Li et al. 2022). Therefore, while an inclusive climate provides a foundation for psychological safety, the direct incentive for the proactive behaviors central to athletic performance is more potently supplied by an innovative climate. Therefore, we presented H3 that:

*Inclusive team climate positively moderates the relationship between mindfulness and proactive behavior* (*a*) *and the relationship between mental toughness and proactive behavior* (*b*).

## 3 Methods

### 3.1 Procedures and Data

An online study has been organized through Questioner Stars (WJX.com) to collect feedback from participants. IRB approval for this study was obtained from the Science and Technology Development Institute of Hainan University. Participants involved in our research are formal athletes who served in competitive sports, such as basketball, soccer, and table tennis, in the Chinese mainland.

We used an existing validated Chinese version of the Mindful Attention Awareness Scale (MAAS) (Deng et al. 2012), and translated the rest of the scales into three steps which contain the organizational innovative climate scale (Newman et al. 2020), the psychological inclusive climate scale (Zahoor et al. 2024), the mental toughness scale (Madrigal et al. 2013), and the proactive behavior scale (Hsiao and Wang 2020). Firstly, to avoid inconsistency in our questionnaire, the first author consulted a professional translator who has a sports psychology background to develop a Chinese version of the scales. Secondly, our last author, as an independent back‐translator, processed the back‐translation, ensuring that the previous translation was linguistically and conceptually equivalent to that of the first author. Thirdly, we have a final reconciliation procedure about our translated questionnaires with the rest of the researchers.

We assigned them online and collected feedback from March to May 2025. For sampling and classification, we verified the number of their athletic certificates and asked them to make a statement. Besides, recruited participants are randomly selected from the online platform, and no sampling‐biased language or questions are used.

Under the guidance of Jak et al. (2021), we calculated the required sample size by using RMSEA‐based approaches. Results show that our sample size is acceptable. Our total number of anonymous feedback is 483, shown in Table 1. The background information and demographic characteristics are shown in Table 1 below. Specifically, gender distribution was balanced, with 238 males (49.3%) and 245 females (50.7%). Regarding work tenure, the largest group comprised individuals with 5–10 years of experience (177, 36.6%), followed by those with 2–5 years (105, 21.7%) and 10–15 years (92, 19.0%). A notable proportion (68, 14.1%) reported having less than 2 years of tenure. Participants were predominantly young, with the vast majority falling within the 19–30 age range (19–25: 198, 41.0%; 26–30: 179, 37.1%). Educational backgrounds varied, with high school diplomas being the most common qualification (228, 47.2%), closely followed by bachelor's degrees (177, 36.6%). Fewer held master's (42, 8.7%) or doctoral degrees (8, 1.7%). In terms of weekly practice commitment, the most frequent range was 11–20 h (149, 30.8%), followed by 6–10 h (110, 22.8%) and 21–30 h (96, 19.9%). Smaller proportions practiced less than 5 h (71, 14.7%) or more than 31 h (57, 11.8%).

**TABLE 1:** Background information and demographic characteristics.

Category	Criteria	*N*	%
Total	/	483	100
Gender	Male	238	49.3
	Female	245	50.7
Tenure	Less than 2 years	68	14.1
	2–5 years	105	21.7
	5–10 years	177	36.6
	10–15 years	92	19.0
	Less than 2 years	41	8.5
Age	Younger than 18	78	16.1
	19–25	198	41.0
	26–30 years	179	37.1
	31–35 years	24	5.0
	Older than 36	4	0.8
Education	Junior below	28	5.8
	High	228	47.2
	Bachelor	177	36.6
	Master	42	8.7
	Doctor	8	1.7
Training	Less than 5 h	71	14.7
	6–10 h	110	22.8
	11–20 h	149	30.8
	21–30 h	96	19.9
	More than 31 h	57	11.8

### 3.2 Measures

#### 3.2.1 Mindfulness

For collecting athletes' mindfulness, we referred to the original work of Brown and Ryan (2003) and an existing validated Chinese scale of Deng et al. (2012) about measuring mindfulness, and applied it in the context of athletes (Guo et al. 2025). The Mindful Attention Awareness Scale (MAAS) was developed with 15 items (Brown and Ryan 2003). We used 7 items of Deng et al. (2012) with a high Cronbach's *α*. Following Fu et al. (2025), we reverse‐coded for some negative questions. Specifically, the items are included: (1) I find myself not concentrating during exercise/training, (2) I may be experiencing an emotion but am not aware of it until sometime later, (3) I find it difficult to maintain focus on what is happening in the present moment during my sport, etc. Responses were made using a 5‐point Likert scale (1 = *little proactivity*; 5 = *very high proactivity*; Cronbach *α* = 0.93).

#### 3.2.2 Proactive Behavior

We asked the athletes to rate their direct report of proactive behavior referred to scales of Griffin et al. (2007) and McCormick et al. (2019) for individual (3 items) proactive behavior. Sample items included: (1) initiated better ways of doing your core tasks, (2) came up with ideas to improve the way in which your core tasks are done, (3) made changes to the way your core tasks are done. Responses were made using a 5‐point Likert scale (1 = *strongly disagree*; 5 = *strongly agree*; Cronbach *α* = 0.85).

#### 3.2.3 Mental Toughness

Mental Toughness has been measured through a simplified 11‐item scale referring to the work of Madrigal et al. (2013), Gucciardi et al. (2015), and Gucciardi et al. (2021). Some of the items we used were listed below: (1) I have an inner arrogance that makes me believe I can achieve anything I set my mind to, (2) I know when to celebrate success but also know when to stop and focus on the next challenge, (3) I have a killer instinct to capitalize on the moment when I know I can win, etc. Responses were made using a 5‐point Likert scale (1 = *strongly disagree*; 5 = *strongly agree*; Cronbach *α* = 0.94).

#### 3.2.4 Inclusive Team Climate

Inclusive team climate has been measured on a 4‐item scale according to Zahoor et al. (2024) and Nishii (2013). Items we used contained (1) team members value the differences that diverse individuals bring to the team as part of the team culture, (2) members highly value constructive suggestions for improvement put forward by other teammates, (3) coaches firmly believe that practical problems can be better solved by considering input from individuals with different positions, ranks, and functions, etc. Responses were made using a 5‐point Likert scale (1 = *strongly disagree*; 5 = *strongly agree*; Cronbach *α* = 0.91).

#### 3.2.5 Innovative Team Climate

We used scales from Newman et al. (2020) and Kim et al. (2024) to collect 4 items for measuring innovative team climate. The items we used included (1) the team's encouragement of members to propose new ideas and solutions, (2) members' innovative experiences and knowledge sharing in sports, and (3) team tolerance for innovation failure. Responses were made using a 5‐point Likert scale (1 = *strongly disagree*; 5 = *strongly agree*; Cronbach *α* = 0.89).

#### 3.2.6 Control Variables

We controlled the individual levels. According to Abrantes et al. (2022) and Slawinska et al. (2025), the athletes' performance, including physical and psychological, was especially affected by their training time. Otherwise, as the formal athletes serve as members of a specific organization, their serving tenure is quite essential to control in our model (Abrantes et al. 2022; Benson et al. 2014). Specifically, we controlled gender, tenure, education level, and training time per week. Supported evidence can also be seen in the research of Podsakoff et al. (1996), Kong et al. (2025), Benson et al. (2014), and Slawinska et al. (2025).

### 3.3 Analytical Strategy

We used the structural equation model following the suggestion of Stanger et al. (2018), Gucciardi et al. (2021), and Fu et al. (2025). Considering the Wayment and Walters (2017) and Kao et al. (2023) research, we used SPSS 29 and the Process Macro of Model 17 to analyze (Hayes 2013). Before the regression analysis, we performed checks for missing values, multicollinearity, skewness, and kurtosis. To avoid common methods bias, we used Herman's single‐factor analysis and adopted the method of controlling potential variables to verify whether there is a significant common method bias (Wen et al. 2023). The confirmatory factor analysis (CFA) has been used to assess the reliability and validity (Kao et al. 2023). Besides, the random sampling is *B* = 5000, and the confidence interval is 95%.

## 4 Results

### 4.1 Bivariate Correlation and Multicollinearity

A bivariate correlation analysis was conducted to examine the relationships between variables. Significant correlations were found between the primary variables. Proactive behavior was significantly positively correlated with mindfulness (*r* = 0.389, *p* < 0.01). Mental toughness was also significantly correlated with proactive behavior (*r* = 0.447, *p* < 0.01). Similarly, inclusive team climate (*r* = 0.409, *p* < 0.01) and innovative team climate (*r* = 0.424, *p* < 0.01) were both significantly correlated with proactive behavior.

The analysis also considered control variables. Gender was not significantly correlated with any of the other control variables, suggesting that gender may not be a confounding factor. Education level showed some significant correlations with mental toughness (*r* = 0.142, *p* < 0.01) and inclusive team climate (*r* = 0.101, *p* < 0.05). Tenure was significantly positively correlated with all five core variables (*p* < 0.01), with coefficients [0.302, 0.425]. Age also showed significant positive correlations with all five core variables (*p* < 0.01), with coefficients [0.190, 0.312]. This may reflect maturity, adaptability, and a positive perception of the work environment brought by tenure or age (Abrantes et al. 2022; Benson et al. 2014). Training is the most widely and relatively strongly associated control variable with the core variables. It exhibits significant positive correlations with all five core variables (*p* < 0.01), with coefficients [0.316, 0.440]. This suggests that training appears to be a highly important background factor that may broadly influence athletes' psychological states and behaviors (Piasecki et al. 2025; Slawinska et al. 2025).

Although the bivariate analysis showed significant correlations between some control variables (such as age, tenure, and training), the VIF values were all below the threshold (VIF < 5.00), indicating that multicollinearity did not affect the reliability of the regression model (Table 2).

**TABLE 2:** Results of bivariate correlation.

Variable	Mean	SD	1	2	3	4	5	6	7	8	9	10
1. Mindfulness	3.148	1.166	1.000									
2. Proactive behavior	3.085	1.284	0.389**	1.000								
3. Mental toughness	2.902	1.046	0.460**	0.447**	1.000							
4. Inclusive team climate	3.023	1.338	0.418**	0.409**	0.469**	1.000						
5. Innovative team climate	2.954	1.416	0.424**	0.424**	0.495**	0.394**	1.000					
6. Gender	0.510	0.500	0.081	0.032	0.061	0.019	−0.041	1.000				
7. Tenure	2.860	1.138	0.407**	0.302**	0.425**	0.378**	0.363**	−0.011	1.000			
8. Age	2.330	0.834	0.286**	0.190**	0.283**	0.312**	0.264**	−0.008	0.746**	1.000		
9. Education level	2.530	0.800	0.036	0.052	0.142**	0.101*	0.069	0.003	0.291**	0.371**	1.000	
10. Training	2.910	1.217	0.392**	0.316**	0.440**	0.379**	0.319**	0.008	0.481**	0.311**	0.101*	1.000

* *p* < 0.05.

** *p* < 0.01.

### 4.2 Reliability and Validity

To ensure the reliability and construct validity of our items, we tested the CFA factor loadings (Byrne 2016; Kline et al. 2011), *p*‐value, Cronbach's *α*, composite reliability (CR), and average variance extracted (AVE), shown in Table 3. According to Cronbach (1951), the scales of measurement are well organized due to the *α* being more than 0.8. Table 3 shows that all our items are significant. As for CR and AVE, Fornell and Larcker (1981) suggested that CRs greater than 0.6 are acceptable. Besides, discriminant validity checks are shown in Table 4. The diagonal values, representing the square root of AVE, are higher than the off‐diagonal correlations, confirming sufficient discriminant validity between the constructs. Based on the above checks, our measurements demonstrate high validity and reliability.

**TABLE 3:** Reliability and construct validity of measurement.

Items	Mean	SD	Factor loading	*p*
Mindfulness (CR = 0.945, AVE = 0.712, *α* = 0.932)				
1	3.06	1.424	0.87	***
2	3.13	1.195	0.77	***
3	3.16	1.406	0.84	***
4	3.28	1.418	0.87	***
5	3	1.535	0.90	***
6	3.15	1.32	0.81	***
7	3.25	1.363	0.84	***
Proactive behavior (CR = 0.909, AVE = 0.770, *α* = 0.847)				
1	3.03	1.558	0.90	***
2	3.12	1.534	0.91	***
3	3.11	1.299	0.82	***
Mental toughness (CR = 0.943, AVE = 0.606, *α* = 0.935)				
1	2.87	1.23	0.74	***
2	2.97	1.185	0.71	***
3	2.93	1.512	0.83	***
4	2.96	1.461	0.84	***
5	2.9	1.479	0.85	***
6	2.89	1.121	0.68	***
7	2.95	1.208	0.71	***
8	2.82	1.568	0.87	***
9	3.03	1.3	0.75	***
10	2.92	1.197	0.71	***
11	2.68	1.431	0.84	***
Inclusive team climate (CR = 0.940, AVE = 0.797, *α* = 0.914)				
1	2.01	1.55	0.92	***
2	2.01	1.29	0.86	***
3	2.02	1.24	0.90	***
4	2.2	1.46	0.89	***
Innovative team climate (CR = 0.930, AVE = 0.816, *α* = 0.885)				
1	2.04	1.46	0.90	***
2	1.86	1.27	0.91	***
3	2.02	1.49	0.90	***

*** *p* < 0.001.

**TABLE 4:** Discriminant validity checks.

Variables	M	PB	MT	Innov	Inclu
M	0.844				
PB	0.417***	0.877			
MT	0.484***	0.491***	0.778		
Innov	0.470***	0.471***	0.538***	0.894	
Inclu	0.445***	0.451***	0.505***	0.438***	0.903

*Note:* The diagonal is the square root of AVE.

Abbreviations: Inclu = innovative team climate; Innov = inclusive team climate; M = mindfulness; MT = mental toughness; PB = proactive behavior.

*** *p* < 0.001.

### 4.3 Confirmatory Factor Analysis and Common Methods Bias

For Common Methods Variance (CMV), Herman's single‐factor analysis results were acceptable, showing the variance explained by the first factor was 40.75% (Williams et al. 2010). The Confirmatory Factor Analysis (CFA) showed that for the baseline five‐factor model (*χ* ^2^ = 374.6, df = 340, *χ* ^2^/df = 1.102, RMSEA = 0.015, NFI = 0.961, RFI = 0.956, CFI = 0.996). We added a method factor of a bi‐factor model (*χ* ^2^ = 311.792, df = 312, *χ* ^2^/df = 0.999, RMSEA = 0.000, NFI = 0.967, RFI = 0.960, CFI = 1.000). Compared to the baseline model and the bi‐factor model, the added method factor yields only limited improvement. We further tested the remaining models shown in Table 5. Thus, a significant common methods bias is not a serious concern in our hypothetical model.

**TABLE 5:** Results of common methods variance.

Model	Variables	*χ* ^2^	df	*χ* ^2^/df	NFI	RFI	RMSEA	CFI
Baseline		374.6	340	1.102	0.961	0.956	0.015	0.996
Bi‐factor		311.792	312	0.999	0.967	0.960	0.000	1.000
Four‐factor	M, PB, MT, Innov+Inclu	1111.692	344	3.232	0.884	0.872	0.068	0.916
Three‐factor	M, PB, MT+Innov+Inclu	2047.591	347	5.901	0.785	0.766	0.101	0.814
Two‐factor	M, PB+MT+Innov+Inclu	2537.46	349	7.271	0.734	0.712	0.114	0.761
One‐factor	M+PB+MT+Innov+Inclu	4550.137	352	12.927	0.523	0.542	0.157	0.542

Abbreviations: Inclu = innovative team climate; Innov = inclusive team climate; M = mindfulness; MT = mental toughness; PB = proactive behavior.

### 4.4 Hypothesis Test

To explore the relationship between athletes' mindfulness and proactive behavior, we adopted two different models: Model 1 (Hayes, Model 17) and Model 2 (Hayes, Model 4), as shown in Tables 6 and 7. The effect of mindfulness on proactive behavior is positive, as shown in Model 2 of Table 7, with a coefficient of 0.22 (*p* < 0.001, SE = 0.05) and a 95% confidence interval of [0.12, 0.32]. In Model 1, which includes moderating variables, the coefficient decreases to 0.11 (*p* < 0.05, SE = 0.05). This result is marginally statistically robust as a lower bound very close to zero. All results remain robust after controlling for gender, tenure, age, education, and training.

**TABLE 6:** Outcome of mediator: mental toughness.

Variables	Model			Model 2		
	*β*	95% CI		*β*	95% CI	
		Lower	Upper		Lower	Upper
Mindfulness	0.26*** (0.04)	0.19	0.34	0.26*** (0.04)	0.19	0.34
Constant	−1.24 (0.18)***	−1.59	−0.90	0.84 (0.18)***	0.49	1.12
Control variables						
Gender	0.08 (0.08)	−0.08	0.23	0.08 (0.08)	−0.08	0.23
Tenure	0.20*** (0.06)	0.09	0.32	0.20*** (0.06)	0.09	0.32
Age	−0.08 (0.07)	−0.22	0.07	−0.08 (0.07)	−0.22	0.07
Education	0.09 (0.05)	−0.02	0.19	0.09 (0.05)	−0.02	0.19
Training	0.20*** (0.04)	0.12	0.27	0.20*** (0.04)	0.12	0.27
Model summary	*R* ^2^ = 0.32***			*R* ^2^ = 0.32***		

*Note:* Std of the coefficient in parentheses.

*** *p* < 0.001.

**TABLE 7:** Outcome of dependent variable: proactive behavior.

Variables	Model 1			Model 2		
	*β*	95% CI		*β*	95% CI	
		Lower	Upper		Lower	Upper
Mindfulness	0.11* (0.05)	0.01	0.21	0.22*** (0.05)	0.12	0.32
Mental toughness	0.23*** (0.06)	0.11	0.35	0.36*** (0.06)	0.25	0.48
Innovative team climate	0.18*** (0.04)	0.10	0.27			
Innovative team climate × mindfulness	−0.11** (0.04)	−0.18	0.04			
Innovative team climate × mental toughness	0.10* (0.04)	0.01	0.18			
Inclusive team climate	0.17*** (0.04)	0.08	0.25			
Inclusive team climate × mindfulness	−0.06 (0.04)	−0.13	0.02			
Inclusive team climate × mental toughness	−0.02 (0.04)	−0.11	0.07			
Constant	3.26*** (0.25)	2.78	3.75	1.02*** (0.24)	0.55	1.48
Control variables						
Gender	0.04 (0.10)	−0.15	0.24	−0.01 (0.10)	−0.21	0.20
Tenure	0.08 (0.07)	−0.06	0.22	0.10 (0.08)	−0.05	0.25
Age	−0.11 (0.09)	−0.29	0.07	−0.05 (0.10)	−0.24	0.13
Education level	−0.01 (0.07)	−0.14	0.12	−0.03 (0.07)	−0.17	0.11
Training	0.06 (0.05)	−0.04	0.15	0.08 (0.05)	−0.02	0.18
Model summary	*R* ^2^ = 0.33***			*R* ^2^ = 0.25***		

*Note:* Std of the coefficient in parentheses.

* *p* < 0.05.

** *p* < 0.01.

*** *p* < 0.001.

Specifically, for H1, the results of Models 1 and 2 in Table 6 are consistent, with the coefficient of mindfulness on mental toughness being 0.26 (*p* < 0.001, SE = 0.04), with an interval [0.19, 0.34]. In Table 7, however, the results differ. In Model 2, the coefficient from mental toughness to proactive behavior is 0.36 (*p* < 0.001, SE = 0.06), with an interval [0.25, 0.48], while in Model 1, this coefficient decreases to 0.23 (*p* < 0.001, SE = 0.06), with an interval [0.11, 0.35]. This confirms our Hypothesis 1 that athletes' mindfulness positively influences proactive behavior through the mediation of mental toughness.

**FIGURE 2:**
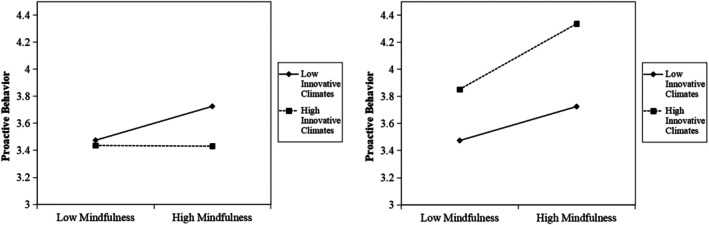
Moderation reverse of innovative team climate.

For H2, as shown in Model 1 of Table 7, the direct effect of innovative team climate on proactive behavior is positive, with a coefficient of 0.18 (*p* < 0.001, SE = 0.04), with an interval [0.10, 0.27]. The interaction term between innovative team climate and mindfulness has a coefficient of −0.11 (*p* < 0.01, SE = 0.04) for proactive behavior, with an interval [−0.18, 0.04]. Therefore, we have no reason to reject that H2a does not hold. However, we found that an innovative team climate weakened the positive effect of mindfulness on proactive behavior. After introducing mental toughness as a mediator, the interaction term between innovative team climate and mental toughness on proactive behavior changed to a significant positive effect, with a coefficient of 0.10 (*p* < 0.05, SE = 0.04), and an interval [0.01, 0.18]. This confirms H2b, which indicates that innovative team climate enhances the positive effect of mental toughness on proactive behavior. The reversed moderation of introducing mental toughness as a mediator is shown in Figure 2 (Rönkkö et al. 2022).

Regarding H3. As shown in Table 7 Model 1, inclusive team climate also has a positive direct effect on proactive behavior, with a coefficient of 0.17 (*p* < 0.001, SE = 0.04), and an interval [0.08, 0.25]. The interaction term between inclusive team climate and mindfulness has a coefficient of −0.06 (*p* > 0.05, SE = 0.04) on proactive behavior. Therefore, we have no reason to reject that H3a does not hold. Similarly, the interaction term between inclusive team climate and mental toughness has a coefficient of −0.02 (*p* > 0.05, SE = 0.04) for proactive behavior, with an interval [−0.11, 0.07]. Therefore, we have no reason to reject that H3b does not hold either.

### 4.5 Extended Findings

Although the hypothesis test showed a lack of significant support for H2a, H3a, and H3b, we got extended findings from the analysis. First, our results didn't support the positive moderation effect in a direct path from mindfulness to proactive behavior (*β* = −0.11). A possible reason for the nonsignificant findings for inclusive climate is the collectivistic context of China. The harmony and conformity of team members might be prioritized and merged into their proactive behavior (Yang et al. 2024). While it provided a reverse when considering mental toughness as a mediator (*β* = 0.10). This explained that mental toughness as a core psychological factor determines the moderating effect of innovative team climate on athletes' proactive behavior, which introduced a complex mechanism of enhancing mindfulness to incentivize proactive behavior (Gucciardi et al. 2021; Madrigal et al. 2013).

Second, we found no significant evidence to support the moderation effect from inclusive team climate (*p* > 0.05 for both direct and indirect). A reason that might explain this is that an inclusive team climate means more collaboration for team members avoiding individuals' risky behavior or intent for proactivity. Otherwise, the positive effect of an inclusive team climate on proactive behavior has been proven to exist, extending the works of Fu et al. (2025), Li et al. (2022), and Nishii and Rich (2014), which indicated that an inclusive climate is positively correlated with mindfulness to athletes' proactive behavior.

Last, we also tested different positions of the two moderators of our hypothesis model in Hayes (2013) model 10, expecting a better model but failed. Results of Model 10 show that there does not exist a moderation effect for innovative team climate and inclusive team climate, which provided us with worse results for our hypothesis. It led us to believe that the moderation of team climate plays its role when mediated through mental toughness. Details of Model 10 are shown in Appendix A.

## 5 Discussion

We tested a moderated mediation model of “mindfulness‐mental toughness‐proactive behavior,” considering the significant moderations of innovative climate. Results showed that: (1) athletes' mindfulness positively influences proactive behavior through the mediation of mental toughness; (2) innovative team climate weakens the positive effect of mindfulness on proactive behavior; (3) innovative team climate enhances the positive effect of mental toughness on proactive behavior. Thus, we conducted several theoretical contributions and practical implications below.

### 5.1 General Incentive of Mindfulness in Proactive Behavior

We identified a general incentive mechanism of formal athletes' mindfulness for proactive behavior. Through the intersection lens of resource conservation theory (Hobfoll 2011) and contextual strength theory (Meyer et al. 2020), we revealed the psychological resource change of mindfulness translating to proactive behaviors. Both Model 1 and Model 2 in Table 6 indicate that mindfulness, through psychological resources accumulation, incentivizes proactive behavior. Although previous studies have explored the incentives of proactive behavior variously (Benson et al. 2014; Li et al. 2024; Macali et al. 2025), research has lacked a general concept of uniformizing differences of proactive behavior across various sports.

We argue that while the incentives of proactive behavior may vary across different sports, mindfulness, as a more internal and deeper factor for athletes, provides insights into the underlying sources driving these behaviors across multiple sports. Differences in athletes' internal mindfulness, combined with specific sport characteristics and other external factors, ultimately lead to changes in proactive behavior. Therefore, unlike previous studies that focused on specific sports contexts (Petrou et al. 2024; Piasecki et al. 2025; Pineau et al. 2014; Shaabani et al. 2020), we further empirically examine the general incentive of mindfulness on proactive behaviors.

### 5.2 Translative Role of Mental Toughness

According to our analysis, mental toughness not only transmits consumption of psychological resources but also translates the incentives from athletes' mindfulness to proactive behavior. This finding bridged the theoretical disconnect between the athletes' mental status and behavioral engagement, explaining proactive behavior through the perspective of pressure (Ticklay and Jones 2024; Zhang et al. 2025).

Results of Models 1 and 2 in Tables 5 and 6 both support a significant positive mediating effect of mental toughness, illustrating a general incentive mechanism through mental toughness. Although previous research has identified motivational factors for proactive behavior across different sport types, such as external stimuli in open‐skill sports and internal stimuli in closed‐skill sports (Macali et al. 2025), as well as personal goals in individual sports and common goals in team sports (Oliver et al. 2010), etc., the common findings of awareness have not yet revealed the translation behind the proactive behavior. Progressively, research has increasingly suggested that mindfulness can enhance athletes' awareness of target behaviors and reduce stress during participation (Shaabani et al. 2020; Terzioğlu and Çakir‐Çelebi 2025); however, the causal mechanisms through which mindfulness translates into proactive behavior within stress mechanisms remain unexplored.

Our study further demonstrates a general concept that athletes' proactive behavior could be enhanced by preserved mindfulness through a pressure perspective of mental toughness. Mindfulness training, such as nonjudgmental attention regulation during high‐pressure scenarios (Fritsch et al. 2024; Shaabani et al. 2020; Ticklay and Jones 2024), should be explicitly linked to mental toughness training (e.g., reframing failures as growth opportunities and managing racing stress) (Gucciardi et al. 2021; Wilson 2018). However, influenced by specific characteristics of different sport types, the contextual differences in mindfulness and mental toughness could lead to diversity in proactive behavior. These potential differences may offer variations in the above incentive mechanism (Ticklay and Jones 2024; Zhang et al. 2025).

### 5.3 Counterintuitive Findings of Innovative Climate and Inclusive Climate

According to the test of H2, we find that the innovative climate, as a moderator, had a significant negative effect on the main effect (*β* = −0.11, *p* < 0.01), while its mediating effect on the relationship between mental toughness and proactive behavior was significantly positive (*β* = 0.10, *p* < 0.05). This mixed moderation pattern means that the innovative climate has an inhibitory effect on the relationship between mindfulness and proactive behavior, but its effect becomes positive when mental toughness serves as a mediating variable. These results suggest that the moderating effect of innovative climate on proactive behavior may differ, given the mediating role of mental toughness.

Specifically, without the translation of mental toughness, athletes in an innovative climate observe stress and pressure directly, losing a buffer or defense against the potential stress caused by peer competition (Wagstaff et al. 2012). Thereby, exerting a negative influence on proactive behavior. Whereas for athletes building a buffer or defense of mental toughness, the pressure from the innovative climate could be transmitted into a peer call of behavioral engagement, promoting additional proactive behavior (Gucciardi et al. 2021). Crucial accordance, innovative team climate should be embedded within an environment where coaches and team members actively endorse risk‐taking and tolerate failures.

As for the inclusive climate, H3 was not supported. An inclusive climate, as a moderating variable, did not significantly activate the incentive mechanism. Although an inclusive climate is believed to provide psychological safety and encourage work behaviors (Nishii and Rich 2014; Yang et al. 2024), our data did not significantly support the hypothesis.

Contrary to our initial expectations regarding an inclusive climate, our predicted results indicate that it exerts a negative moderating effect on both direct (*β* = −0.06, *p* > 0.1) and indirect effects (*β* = −0.02, *p* > 0.1). This result may be related to the core context of an inclusive climate. While an inclusive climate provides psychological safety by reducing external pressure, it may not sufficiently provide bonuses for extra efforts from its members. In the context of collective teamwork, an inclusive climate encourages team members' intentions to collaborate and share wins with others. However, the reasons for engaging in proactive behavior are to gain more than others from its uniqueness and pioneering, which in an inclusive climate may fall short of individuals' expectations. It indicates that coaches should thus explicitly reward the extra efforts from the athletes—such as athlete‐proposed training modifications or opponent analysis—through formal recognition (e.g., innovation points in team evaluations) (Olympiou et al. 2008).

Although our study proposes this mechanism, alternative explanations may still exist. For example, factors such as team leadership style (Arthur and Bastardoz 2020), member trust (Wang et al. 2023), or individual self‐efficacy (Saville and Bray 2016) may play significant roles in the influence of the different climates. Furthermore, cultural background and individual differences among team members may also affect the moderating role in different contexts, such as the collectivistic context of China (Yang et al. 2024).

### 5.4 Limitations and Future Directions

Despite contributions, our study carried inherent limitations to be handled in further research. First, the cross‐sectional design restricts time inferences regarding the dynamic relationships among mindfulness, mental toughness, and proactive behaviors. The time change and period design of our research may influence the preference of questionnaire participants. This may influence our analysis. Although our model aligns with theoretical frameworks, the possibility of reverse causality—such as proactive behaviors reinforcing mental toughness—cannot be completely ruled out. Further research could investigate such backward causal inference. Additionally, mental toughness on self‐reported measures introduces risks of common method bias, despite our statistical controls suggesting minimal impact (Podsakoff et al. 2003). Future research may use longitudinal or experimental designs to eliminate such methodological bias—for instance, tracking athletes' behavioral shifts before and after mindfulness interventions—and integrate objective indicators (e.g., coach evaluations of tactical innovation frequency) to triangulate proactive behavior assessments.

Future studies should explore contextual and individual‐level moderators beyond team climate. For example, coaching leadership styles may interact with innovative climates to further amplify mental toughness effects, a nuance our model did not capture (Benson et al. 2014; Kao et al. 2023). Cross‐cultural validation is also critical. Our China‐centric sample limited generalizability to collectivist contexts, whereas athletes in other cultures might respond differently to inclusive climates (Barnett et al. 2019). Finally, investigating neuropsychological mechanisms could uncover biological pathways linking resource conservation to proactive behaviors (Nien et al. 2020). Such multi‐method approaches would deepen the theoretical integration of organizational psychology and sports science while refining practical interventions.

## Funding

This work was supported by Research Grant Program for Educational Teaching Reform in Hainan Higher Education Institutions (Hnjg2023‐36) and Hainan University Education Teaching Reform Research Project (lhdjy2331).

## Ethics Statement

Ethics approval for this study was obtained from the Science and Technology Development Institute of Hainan University. All participants were recruited through voluntary and noncoercive means. Before participating in the survey, all participants were provided with detailed information about the purpose of the study, the procedures involved, the potential risks and benefits, and the confidentiality measures in place to protect their personal information.

## Conflicts of Interest

The authors declare no conflicts of interest.

## Data Availability

To protect participants' personal privacy, the data availability are as follows: (i) The raw data are available upon reasonable request, and (ii) that any shared dataset is strictly anonymized and contains no direct identifiers or re‐identifiable information, in accordance with the approved ethics protocol and informed consent.

## Appendix A Table A1

**TABLE A1:** Results of multiple collinearity analysis.

Model	Unstandardized		Standardized		*p*	Collinearity statistics	
	*β*	SD	*β*	*t*		Tolerance	VIF
Constant	0.873	0.23		3.795	< 0.001		
Mindfulness	0.135	0.052	0.123	2.589	0.01	0.654	1.528
Mental toughness	0.225	0.062	0.183	3.629	< 0.001	0.578	1.73
Inclusive team climate	0.166	0.045	0.173	3.716	< 0.001	0.68	1.471
Innovative team climate	0.174	0.042	0.192	4.11	< 0.001	0.673	1.485
Gender	0.039	0.099	0.015	0.396	0.692	0.98	1.02
Tenure	0.072	0.073	0.063	0.974	0.331	0.347	2.881
Age	−0.093	0.092	−0.061	−1.011	0.312	0.41	2.436
Education	−0.016	0.067	−0.01	−0.24	0.81	0.849	1.178
Training	0.052	0.049	0.049	1.053	0.293	0.668	1.497

*Note:* Dependent variable: proactive behavior.

To explore the relationship between athletes' mindfulness and proactive behavior, we also adopted another different model: Model 3 (Hayes 2013, Model 10), as shown in Table A2.

**TABLE A2:** Outcome of mediator mental toughness.

Variables	Model 3		
	*β*	95% CI	
		Lower	Upper
Mindfulness	0.14*** (0.04)	0.07	0.22
Innovative team climate	0.19*** (0.03)	0.13	0.25
Innovative team climate × mindfulness	0.04 (0.03)	−0.02	0.09
Inclusive team climate	0.16*** (0.03)	0.09	0.22
Inclusive team climate × mindfulness	−0.01 (0.03)	−0.06	0.04
Constant	2.03*** (0.17)	1.70	2.36
Control variables			
Gender	0.12 (0.07)	−0.03	0.26
Tenure	0.14** (0.05)	0.04	0.25
Age	−0.10 (0.07)	−0.24	0.03
Education	0.09 (0.05)	−0.01	0.18
Training	0.14*** (0.04)	0.07	0.21
Model summary	*R* ^2^ = 0.42***		

*Note:* Std of the coefficient in parentheses.

** *p* < 0.01.

*** *p* < 0.001.
